# Under my umbrella: Rating scales obscure statistical power and effect size heterogeneity

**DOI:** 10.3758/s13428-025-02879-w

**Published:** 2025-11-24

**Authors:** Jens H. Fünderich, Lukas J. Beinhauer, Frank Renkewitz

**Affiliations:** 1 University of Erfurt, Nordhäuser Str. 63, 99089 Erfurt, Germany; 2 Ludwig-Maximilians-Universität München, Munich, Germany

**Keywords:** Rating scales, Heterogeneity, Power, Error, Meta-science

## Abstract

Data from rating scales underlie very specific restrictions: They have a lower limit, an upper limit, and they only consist of a few integers. These characteristics produce particular dependencies between means and standard deviations. A mean that is a non-integer, for example, can never be associated with zero variability, while a mean equal to one of the scale’s limits can only be associated with zero variability. The relationship can be described by umbrella plots for which we present a formalization. We use that formalization to explore implications for statistical power and for the relationship between heterogeneity in unstandardized and standardized effect sizes. The analysis illustrates that power is not only affected by the mean difference and sample size, but also by the position of a mean within the respective scale. Further, the umbrella restrictions of rating scales can impede interpretability of meta-analytic heterogeneity. Estimations of relative heterogeneity can diverge between unstandardized and standardized effects, raising questions about which of the two patterns of heterogeneity we would want to explain (for example, through moderators). We reanalyze data from the Many Labs projects to illustrate the issue and finally discuss the implications of our observations as well as ways to utilize these properties of rating scales. To facilitate in-depth exploration and practical application of our formalization, we developed the *Shiny Umbrellas* app, which is publicly available at https://www.apps.meta-rep.lmu.de/shiny_umbrellas/.

## Introduction

How generous was the customer's tip? Was it wrong of the boss to discourage unionizing? How much do you agree with the previous statement? These types of questions are ubiquitous in psychological and social science research. Participants are often asked to respond to such questions on rating scales, tying their answers to the characteristics of these measures. A typical rating scale has an upper and a lower bound, consists only of integers, and is applied in a sample of finite size. These features of the scale affect the aggregates calculated from the participants’ responses. For an illustrative example, we assume a three-point scale from 1 to 3 and responses from two participants. There are only five observable means under these conditions: $\overline{x }\in \{1, 1.5, 2, 2.5, 3\}$. Further, each mean implies different restrictions on the associated variability: Means 1 and 3, the upper and lower bounds, can only coincide with zero variability, while 1.5 and 2.5, the non-integers, cannot coincide with zero variability. Thus, means and standard deviations do not vary independently of each other when they are aggregations of data from a rating scale. The literature on research misconduct exploits these features of rating scales to detect errors in scientific reporting, using techniques like the GRIM test (Brown & Heathers, 2017), the GRIMMER test (Anaya, 2016), and SPRITE (Heathers et al., 2018). Brown and Heathers (2017) create a scatter plot with all combinations of means and standard deviations for a five-point scale and a sample size of 10 with the former on the *x*-axis and the latter on the *y*-axis. The combinations of means and standard deviations scatter within the shape of an umbrella, and the authors appropriately refer to these as umbrella plots. Taylor et al. (2023) notice similar patterns in data from norming studies in which large numbers of participants evaluate items on rating scales. They conclude that standard deviations and variances from rating scales are inadequate to compare inter-rater agreement across samples because of the dependency on the respective mean rating. Samples with average ratings close to the center of the scale can indicate much less agreement than those at the scale’s limits.

**Fig. 1:**
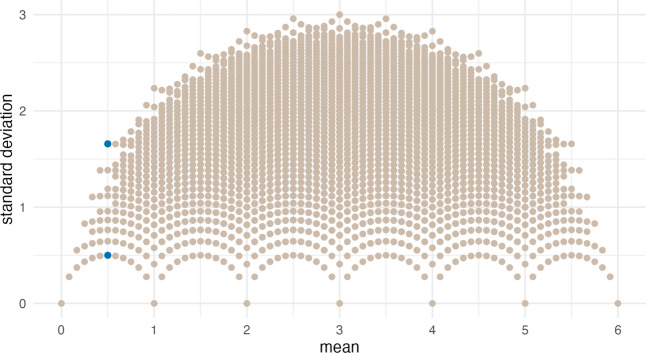
The umbrella plot with all combinations of means and sample standard deviations for a seven-point scale and $n = 12$. The blue dots represent the samples that have the smallest and largest standard deviation for a mean $\overline{x }=0.5$ on the given scale and sample size. We provide an openly available and simple version of this simulation to explore in a Shiny App at https://www.apps.meta-rep.lmu.de/shiny_umbrellas/ in the tab *Discrete Umbrella*

The umbrella plot (Fig. 1) describes a parameter space to which means and standard deviations obtained from rating scale data are restricted. Here, we investigate the implications of these restrictions for the application of parametric analyses to rating scales. There is, of course, a long-standing discourse around the application of ordinal data to parametric analyses—for summaries see, for example, Lalla (2017) or Kampen and Swyngedouw (2000). Relatedly, there is also a broad literature on the robustness of parametric statistics to violations of their underlying assumptions, like normality, that is related to our work (e.g., Hsu & Feldt, 1969; Mircioiu & Atkinson, 2017; Norman, 2010). However, this literature typically focuses on comparing nominal and effective power or type 1 error (e.g., Norman, 2010; Van Hecke, 2012), or the mapping of latent traits to discrete scales from a measurement perspective (e.g., Andrich, 1978; Koch, 1983; Samejima, 1969).

In contrast, we explore how the restrictions of rating scales constrain the possible combinations of means and standard deviations, and how they affect parametric statistics. In this article, we (1) formalize these restrictions on rating scales represented in the umbrella and explore implications for the interpretability of (2) statistical power of *t*-tests and of (3) meta-analytic effect heterogeneity. For our investigation into effect heterogeneity, we use three meta-analytic data sets from Many Labs 1 (Klein et al., 2014) and Many Labs 2 (Klein et al., 2018). Note that we do not argue for an alternative model or for specific assumptions around latent constructs behind the data. Rather, we point to consequences of the decision to apply common procedures like *t*-tests or meta-analyses to data collected on a rating scale. We take the observation of the umbrella as a starting point to our exploration, as it captures not just the fact that the scale is limited (for a comprehensive analysis of ceiling and floor effects, see Šimkovic & Träuble, 2019), but also the dependency between means and standard deviations. We introduce these implications in the context of our, possibly narrow, definition of a rating scale as having an upper and a lower bound, consisting only of integers, and being applied in samples of finite size. The *General Discussion* revisits this definition, and relates our findings to scales and measures with related characteristics, such as observations of rare events.

## Umbrella plots: Formalizing the dependency between means and standard deviations

Heathers et al. (2018) visualize the possible combinations of means and standard deviations for data from rating scales in a scatter plot. When means are assigned to the *x*-axis and standard deviations to the *y*-axis, the resulting pattern closely resembles that of an umbrella (without its handle), centered around the mean of the scale. We created all combinations of integers from a seven-point scale for a sample size $n=12$ using the *gtools* package (Warnes et al., 2023) and report the respective sample means and standard deviations in Fig. 1. The curvature at the top of the umbrella implies that means around the scale’s center can coincide with much larger standard deviations than means at its extremes. A mean can only assume the lowest or highest integer of a scale if all participants select that value. At both of these extremes, there is no variance in the data—the standard deviation is zero. Means and standard deviations are not independent of each other when they are calculated on data from a rating scale.

We can formalize the relationship between means and standard deviations by focusing on the minimally and maximally achievable standard deviation per mean (each point on the *x*-axis). To illustrate the idea, we assume a mean $\overline{x }=0.5$, $n=12$, and the same seven-point scale as in Fig. 1. Table 1 reports hypothetical individual participant data (IPD) for the lowest and highest possible variation for the respective mean. By assigning individual responses in equal amounts to the two closest integers, we attain the lowest possible standard deviation, ${s}_{min}$, for our example mean $\overline{x }=0.5$. The respective largest possible standard deviation, ${s}_{max}$, is attained by assigning the responses to the smallest and largest integer of the scale.

**Table 1:** Hypothetical individual participant data

$i$	1	2	3	4	5	6	7	8	9	10	11	12
${s}_{min}$	0	0	0	0	0	0	1	1	1	1	1	1
${s}_{max}$	0	0	0	0	0	0	0	0	0	0	0	6

The columns 1 to 12 each represent a participant, ${n}_{i}$. ${s}_{min}$ and ${s}_{max}$ represent the samples with the smallest and largest standard deviation at $\overline{x }=0.5$

The combinations of means and standard deviations resulting from the two data sets in Table 1 are highlighted in blue in Fig. 1. Calculating both sample standard deviations, defined as $s=\sqrt{\frac{\sum_{i}^{n}{({x}_{i}-\overline{x })}^{2}}{n}}$, results in ${s}_{min}=0.5$ and ${s}_{max}=1.658$. Notably, the data sets we create to minimize and maximize the standard deviation are binary, respectively consisting only of zero and one additional integer. We can describe binary data via Bernoulli distributions, which have the particular property that their standard deviation, calculated as $\sqrt{p(1-p)}$, only depends on the expected value of the distribution (or vice versa). The expected value, $p$, is the probability for the event $X=1$, or the proportion of participants who responded with that value, so that $\mathrm{Pr}\left(X=1\right)=p=1-\mathrm{Pr}\left(X=0\right)$. For the example of ${\sigma }_{min}$ in Table 1, $p$ is equivalent to the arithmetic mean, $\overline{x }=p=0.5$, because all responses are either 0 or 1. Therefore, the minimum standard deviation according to the Bernoulli distribution is

$${s}_{min}=\sqrt{p\left(1-p\right)}=0.5.$$

For ${\sigma }_{max}$, we calculate the expected value of the Bernoulli distribution as $p=\frac{\overline{x}}{k }=\frac{0.5}{6}=0.083$, where $k$ is the number of thresholds of the scale, $k={x}_{max}-{x}_{min}=6-0=6$. For a scale that starts at zero, the number of thresholds is identical to the largest integer of the scale. Dividing the arithmetic mean $\overline{x }$ from the rating scale responses by the number of thresholds scales it to $p$, which lies between 0 and 1. Thus, to calculate the maximum standard deviation, we scale $\overline{x }$ to $p$, calculate the Bernoulli variance, re-scale it to the original units (by multiplying it by the square of $k$), and take its square root:

$${s}_{max}=\sqrt{{k}^{2}p(1-p)}=1.658.$$

The sample standard deviations which we calculated based on the individual responses in Table 1 are identical to the minimum and maximum standard deviations based on the Bernoulli estimate. For a similar formalization of variances for rating scales that requires the disaggregated data, see Brown and Simcock (2023).

**Fig. 2:**
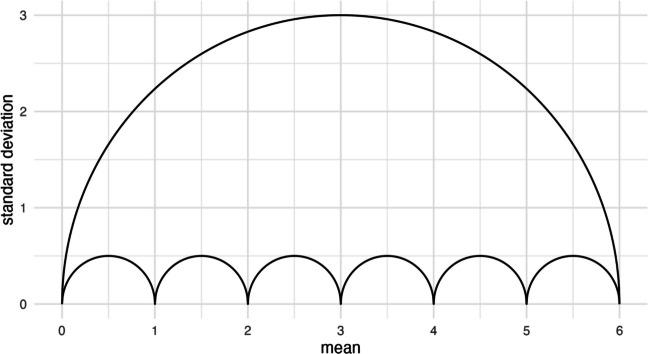
The *x*-axis represents means and the *y*-axis sample standard deviations from rating scales. The plot depicts the outline of the umbrella for a scale from $0$ to $6$. This umbrella outline can be explored in the *Shiny Umbrellas* app, for example in the *Error Checking* tab at https://www.apps.meta-rep.lmu.de/shiny_umbrellas/

We can repeat this procedure for all means across the length of a specific scale to outline the possible combinations of means and standard deviations for that scale. Figure 2 is an example of this type of umbrella plot, which depicts the restrictions independent of the sample size. Any data point above or below the umbrella is impossible. The sample size determines how many points lie within that umbrella and which means can be assumed between the integers. At $n = 2$, for example, $\overline{x } = 0.5$ is the only integer between $0$ and $1$, and because there is only one solution for attaining that integer, ${s}_{min}={s}_{max}=0.5$. Note that this umbrella describes the restriction for the sample standard deviation that is calculated with $n$, not $n-1$, in its denominator. The closer a data point is to the bottom of the umbrella (low on the *y*-axis), the smaller the spread of responses is across the scale, while closeness to the upper edge, especially of means towards the center of the scale, implies more polarized results.

Observations in which values stack up on either end of a limited measure are often classified as floor or ceiling effects. We do not need the umbrella to demonstrate that these will be associated with smaller standard deviations, and we know that measures of dispersion like the standard deviation have a lower bound of zero. However, the Bernoulli distribution formalizes this relationship and additionally allows us to identify the upper bound of variability for that scale at its center. A measure cannot express variability beyond that point—the sample standard deviation of data from a seven-point scale, for example, can never be larger than 3.

While this relationship is helpful for detecting error or fraud, it can obscure the interpretability of parametric statistics. A standardized mean difference, for example, relates the unstandardized effect to the pooled standard deviations of two experimental conditions, which are restricted as outlined (e.g., in Fig. 2). If the standardized mean difference is affected by the limitations of the scale, so is statistical power for a *t*-test, as both relate the unstandardized effect to the variability within the experimental conditions. The following section explores restrictions to statistical power of data obtained from a rating scale through our formalization of the umbrella.

## Statistical power

Here, we describe implications of the umbrella for nominal statistical power, focusing on experimental comparisons between two groups. When researchers test for an effect within a single such experiment, they often apply some form of *t*-test. The generalization of the *t*-test by Welch (1947) allows the population variances (and standard deviations) of the groups to differ and has been proposed as the default *t*-test for psychological research due to its robustness (Delacre et al., 2017). Means from rating scales that are not identical—all nonzero mean differences—are by design likely to produce unequal variances, as the umbrella demonstrates. Therefore, we explore the implications for statistical power of Welch’s *t*-test in this section. Note that we focus on nominal power here and that effective power could (and often will) deviate from it, as the underlying distributions are usually non-normal (e.g., Cribbie & Keselman, 2003; Delacre et al., 2017; Sawilowsky & Blair, 1992). The work of Heeren and D'Agostino (1987) complements our analyses especially well, as they investigated deviations between nominal and effective power of independent samples *t*-tests for short rating scales and small samples by creating all possible individual participant data distributions.

First, we generally describe how rating scales produce an association between the location of the group means on the scale and the nominal power of the test. Subsequently, we demonstrate that association for a fixed unstandardized effect on a seven-point scale. To simplify our notation and language throughout this article, we categorize the two experimental conditions of a data set as control and treatment groups. There are other types of two-group designs, of course, and we will later briefly touch on the role of the experimental design within a meta-analytic context.

### Rating scales and nominal statistical power

How could statistical power be affected by the relationship between means and standard deviations depicted by the umbrella? To calculate the test statistic of Welch’s *t*-test, we divide the effect size, the mean difference, by its standard error. In case of equal sample sizes, the standard error of the mean difference is attained by taking the square root of the sum of variances from the control and treatment group means:

$${s}_{{\overline{x} }_{t}-{\overline{x} }_{c}}=\sqrt{{s}_{{\overline{x} }_{c}}^{2}+{s}_{{\overline{x} }_{t}}^{2}}.$$

The variances for the group means are ${s}_{{\overline{x} }_{c}}^{2}=\frac{{s}_{c}^{2}}{{n}_{c}}$ and ${s}_{{\overline{x} }_{t}}^{2}=\frac{{s}_{t}^{2}}{{n}_{t}}$, respectively, where ${s}_{c}^{2}$ and ${s}_{t}^{2}$ are the unbiased sample variances. A mean difference calculated from two groups close to a scale’s center can assume much larger standard deviations and standard errors than the same effect size with both groups closer to one of the scale’s limits. As a mean approaches a scale limit, ${s}_{max}$ decreases. Power is the smallest when both group means are at ${s}_{max}$. If ${s}_{max}$ decreases, the smallest possible statistical power increases. The exact same (unstandardized) effect and sample size can be associated with different ranges of statistical power in hypothesis tests if group means are at different positions within the scale. In the following paragraphs, we demonstrate these restrictions to power for the case of a seven-point rating scale.

### The range of nominal power of a seven-point scale

**Fig. 3:**
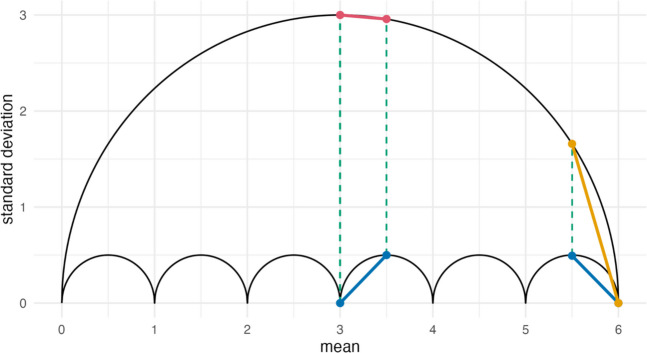
The plot depicts the outline of the umbrella for a scale from $0$ to $6$. Additionally, it depicts group means and standard deviations of four replications. The red line has the highest and the blue line the lowest possible ${\sigma }_{pooled}$ for any $MD=0.5$ with a control mean ${\overline{x} }_{c}=3$ and a treatment mean ${\overline{x} }_{t}=3.5$. The yellow line has the highest, and the blue line the lowest possible ${s}_{pooled}$ for any $MD=0.5$ with a control mean ${\overline{x} }_{c}=5.5$ and a treatment mean ${\overline{x} }_{t}=6$

The relative position of a group mean within the limits of a scale is directly tied to a specific range of standard deviations, and therefore of statistical power (assuming a constant sample size). A replication with a mean difference $MD=0.5$ has a larger range of potential power if it is positioned at the center of the scale rather than at one if its extremes. Figure 3 depicts four hypothetical replications with identical effects $MD=0.5$. Two of them are at the scale’s center with means ${\overline{x} }_{c}=3$ and ${\overline{x} }_{t}=3.5$, and two at its upper limit with means ${\overline{x} }_{c}=5.5$ and ${\overline{x} }_{t}=6$. The replication represented in red has the largest possible standard deviations for its respective means. Our formalization of the umbrella outline allows us to calculate them and, therefore, the lowest possible power for $MD=0.5$ in that interval (per sample size). If we repeat that procedure for the blue replication within the same interval, we receive the range of statistical power for $MD=0.5$ with ${\overline{x} }_{c}=3$ and ${\overline{x} }_{t}=3.5$ on a rating scale from 1 to 7. These hypothetical replications demonstrate the implied range of nominal power at the respective position within the scale, rather than reflecting probable experimental outcomes.

#### Power analyses

We calculate statistical power for sample sizes from $n=24$ to $n=720$. The group sizes, ${n}_{c}$ and ${n}_{t}$, are multiples of $12$, as this is the smallest sample size for which ${s}_{max}$ can be assumed at ${\overline{x} }_{c}=5.5$ (see Table 1) on this seven-point scale. We calculate the standard deviations for the respective means from Fig. 3 using the Bernoulli formalization and use these to calculate statistical power of Welch’s *t*-test with the package *MKpower* (Kohl, 2024) in R (R Core Team, 2021).

**Fig. 4:**
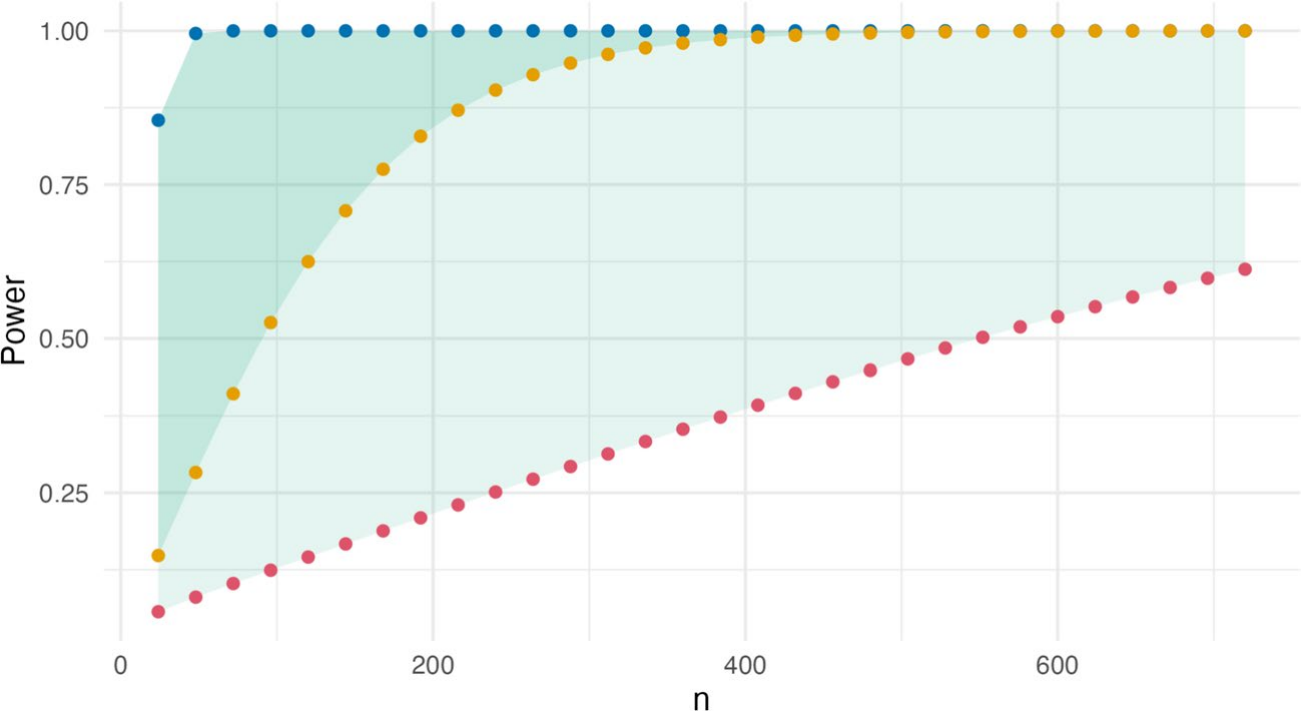
The plot depicts the combined sample size of the experimental conditions, $n={n}_{c}+{n}_{t}$, on the *x*-axis and the resulting power on the *y*-axis. Blue dots represent results for the two blue lines in Fig. 2. Yellow dots represent the results for the yellow line in Fig. 2, which is at the limit of the scale and the red dots those for the red line at its center

Figure 4 reports statistical power of Welch’s *t*-test (*y*-axis) for the respective sample size (*x*-axis). The studies at the bottom of the umbrella of Fig. 3 are represented as blue dots in Fig. 4. These result in statistical power of about $1$ or $100\%$ for all $n\ge 48$. While these upper limits of power are identical for the two intervals, the differences at the lower limits of potential power are quite large. Any effect $MD=0.5$ in the interval from $x=5.5$ to $x=6$ with a sample size $n=192$ has about $80\%$ power or more (yellow points). Conversely, the lowest possible power for the interval from $x =3$ to $x=3.5$ for the same $n=192$ is still below $25\%$.

Rating scales introduce a dependency between the effects’ position on the scale and the minimal statistical power to find an effect. In a randomized design with an untreated control condition, we can use the control group mean as a (possibly noisy) estimate for the respective population’s baseline of the dependent variable. If this baseline varies across populations, so could the control group means. Our results imply that the power of a test applied to a rating scale is not only related to the size of the effect, but also to the respective baseline estimate. If we sample from a population with a baseline close to one of the extremes, power is likely to be higher than it is for a population with a baseline close to the scale’s center.

**Fig. 5:**
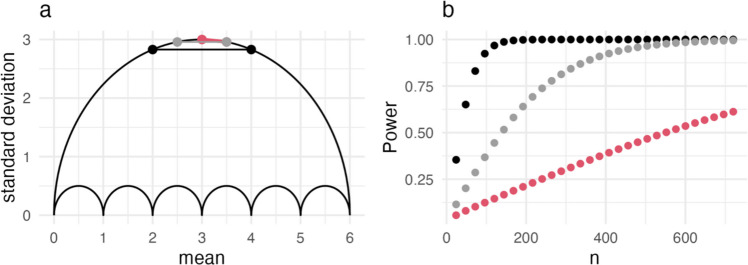
Plot **a** depicts the outline of the umbrella for a scale from $0$ to $6$. Additionally, it contains three hypothetical study results with $MD=0.5$ (red), $MD=1$ (gray), and $MD=2$ (black). Plot **b** presents the results of power analyses for these three effects with sample sizes $n=24$ to $n=760$ (for both groups combined). The *Shiny Umbrellas* app contains the *Power* tab in which we implemented a version of this analysis that allows the user to specify a scale and mean difference to create the umbrella, as well as nominal power at different sample sizes: https://www.apps.meta-rep.lmu.de/shiny_umbrellas/

So far, we have kept the unstandardized effect constant and plotted statistical power at different positions within the scale. Figure 5a depicts three effects with $MD=0.5$, $MD=1$, and $MD=2$, each located at the umbrella’s upper outline to maximize the standard deviations. Thus, any other $MD=0.5$, $MD=1$, and $MD=2$ on a seven-point rating scale (for group sizes ${n}_{c}\ge 12$ and ${n}_{t}\ge 12$) would have larger statistical power than what we see in Fig. 5b. For any effect $MD\ge 2$ (black), power is close to $100\%$ if the combined sample size is $n\ge 96$. A priori power analyses require us to make assumptions around both the unstandardized effect and the standard deviations. A lack of prior knowledge or of reasonable assumptions can drive scientists into the arms of conventions and their potential pitfalls. As a more informed alternative, the restrictions of the rating scale allow us to identify the largest possible standard deviations for an assumed mean difference. Considering such restrictions in a priori power calculations can contribute to an appropriate allocation of resources. For example, we could set that a treatment is only of interest to us if the effect is $MD \ge 1$ on a seven-point scale (gray in Fig. 5). Since the pooled standard deviation is restricted to ${s}_{pooled}<3$, the standardized effect will always be $d>0.33$. For this example, assuming a smaller standardized effect in an a priori power analysis would needlessly inflate the required sample size.

The location of an effect, or that of the experimental conditions, within the umbrella affects the minimal statistical power of a *t*-test. Standardized mean differences, $d$, are affected similarly: Fig. 5a depicts three mean differences that assume the smallest respective $d$ for that scale. The larger an unstandardized effect is on a rating scale, the smaller the associated ${s}_{pooled}$ and the larger the standardized effect, $d$. If effects in a data set create a pattern similar to that in Fig. 5a, for example, the relative differences between the effects is smaller in $MD$ than in $d$. Thus, the choice of effect size could affect our impression of the consistency across effects. This consistency is typically evaluated as heterogeneity, the variability of effects after correcting for sampling error, by applying a meta-analysis (e.g., Borenstein et al., 2010). A dependency of our evaluation of effect heterogeneity on the choice of effect size would raise important concerns: How strongly do the results diverge? Which heterogeneity is relevant to my interpretation of the effects? Which is the more appropriate effect size to identify relevant moderators? In the next section, we explore how rating scales induce differences between heterogeneity results for standardized effects and their unstandardized counterparts.

## Heterogeneity

While we assume most verbal hypotheses relate to unstandardized effects (e.g., the mean of the experimental condition is assumed to be larger than that of the control condition), meta-analyses, even of direct replications, commonly aggregate standardized effects. We also assume that choosing standardized effects within a meta-analytic context is often either habitual or pragmatic, for example, because it allows for some intended comparison. But rating scales can induce systematic differences between unstandardized and standardized effects. In this section, we first establish how the properties or restrictions of the rating scale relate to differences between unstandardized and standardized effects and subsequently explore meta-analytic data from Many Labs 1 (Klein et al., 2014) and Many Labs 2 (Klein et al., 2018) for such differences. We conclude with remarks on designs that make deviations between the two effect size measures more likely.

This section illustrates the consequences of meta-analyzing data aggregated as unstandardized or standardized mean differences, as this approach is still quite common, even when better alternatives are viable. Nonetheless, we want to point out that approaches like the bivariate meta-analysis of group means (McShane & Böckenholt, 2019), or one-stage meta-analysis (e.g., Riley et al., 2008; van Aert, 2022), are often preferable, when applicable. Moreover, there is a large body of literature that suggests standardization should only be applied when it is absolutely necessary due to the interpretational pitfalls of standardized effects and advantages of unstandardized effects (e.g., Baguley, 2009; Bond et al., 2003; Greenland et al., 1986; Tukey, 1969; Wilkinson, 1999).

### Illustrating the argument

The standardized mean difference $d=\frac{MD}{{s}_{pooled}}$ is a ratio of the mean difference, $MD$, and the pooled standard deviation, ${s}_{pooled}$, that is a weighted average of the standard deviations of both groups. Thus, in the case of data from a rating scale, we standardize with an ${s}_{pooled}$ that is affected by the restrictions described by the umbrella.

**Fig. 6:**
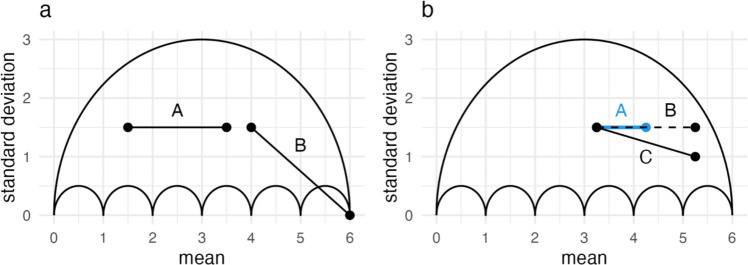
The plots depict the outline of the umbrella for a scale from $0$ to $6$. Additionally, they contain hypothetical study results

For demonstrative purposes, we assume an original study where the dependent variable was measured on a rating scale from 0 to 6 in two experimental conditions. Both conditions have the same sample size, which we assume to be large enough for us to ignore sampling error for now (and return to it in our analyses of multi-lab data). The unstandardized effect is$MD=2$, represented by line A in Fig. 6a. Control and treatment groups produce the same variance, resulting in${s}_{pooled}=1.5$, and a standardized effect of$d=1.33$. Line B in Fig. 6a represents a hypothetical replication of the same design based on a sample of the same size. $MD=2$ is identical to line A, but the standardized effects $d$ differ. The treatment group mean of line B is at the scale’s limit, where no variation is possible, resulting in ${s}_{pooled}=\sqrt{1.125}$ and $=\frac{2}{\sqrt{1.25}}=1.89$. The unstandardized effects are homogeneous; they are in fact identical. All heterogeneity in the standardized effects is introduced by that of the standard deviations in such a scenario. Figure 6a depicts an illustrative (and extreme) example of the fact that unstandardized and standardized effects regard different information. If ${s}_{pooled}$ is heterogeneous across replications, the distributions of *MD* and *d* may diverge, especially if $MD$ and ${s}_{pooled}$ have a nonzero correlation.

Figure 6b depicts a second example in which line A represents the original study and lines B and C are two replications of the same design. The control groups for all three are as homogeneous as they could be: They share the same mean and standard deviation. Both replications are associated with the same unstandardized effect $MD=2$, which is twice as large as the original A. The standardized effects, on the other hand, differ between the two replications. Replication B results in the same standardized effect as that of the original A from Fig. 6a, ${d}_{B}=1.33$, and replication C results in ${d}_{C}=\frac{2}{\sqrt{\frac{{1.5}^{2}+{1}^{2}}{2}}}=1.57$. In this scenario, the choice of effect size affects the observed relation between effects. Further, we believe that there are many scenarios that make replication C a probable outcome if a replication of study A results in a larger effect. We know from our formalization that the upper outline of the umbrella represents samples where all the participants’ responses are at the scale limits. The distance between a data point and that edge provides us with an intuition of how polarized the results are. The treatment group of Replication B in Fig. 6b is quite close to that outline relative to the original A and replication C. If we do not assume that responses get increasingly polarized with an increase in effect size, replication C seems like the more plausible scenario. Therefore, the associated standard deviations decrease as mean differences increase, introducing covariation between the two that could affect heterogeneity in standardized effects. Replication C, the scenario in Fig. 6a, and that of Fig. 5a are examples for which standardized effects $d$ are less consistent than the respective $MD$. If these differences are also reflected in meta-analytic results, they can affect our evaluation and explanation of effect heterogeneity. In the following section, we formalize a comparison between heterogeneity of $MD$ and $d$.

### Formalizing the argument

We cannot directly compare absolute heterogeneity $\tau$, the standard deviation of true effects, of $MD$ and $d$. The former reports the mean difference in units of the scale and the latter in terms of standard deviations. But the coefficient of variation, $CV$, is a relative heterogeneity measure that standardizes $\tau$ on the mean of the distribution, $\upmu$. It allows us to compare relative heterogeneity of unstandardized and standardized mean differences, ${CV}_{MD}$ and ${CV}_{d}$. Renkewitz et al. (in preparation) formalize standardized mean differences as a ratio distribution: $d$ is the ratio of the random variables $MD$ and ${\sigma }_{pooled}$. They present a formalization for relative heterogeneity in $d$ for the coefficient of variation:

1 $${CV}_{d}=\sqrt{{CV}_{MD}^{2}+{CV}_{{\sigma }_{pooled}}^{2}-2{r}_{MD,{\sigma }_{pooled}}{CV}_{MD}{CV}_{{\sigma }_{pooled}}}$$

with the coefficients of variation for the unstandardized and standardized effects and for the pooled standard deviations—${CV}_{MD}$, ${CV}_{d}$, and ${CV}_{{\sigma }_{pooled}}$, respectively. ${r}_{MD,{\sigma }_{pooled}}$ is the correlation between the unstandardized mean differences and the pooled standard deviations. For cases of homogeneous (nonzero) $MD$ (see Fig. 6a for an example), we can simplify Eq. (1) to

2 $${CV}_{d}={CV}_{{\sigma }_{pooled}}$$

All heterogeneity in $d$ is induced by the pooled standard deviations. In a scenario like this, any moderators introduced to explain effect heterogeneity would need to be associated with the variability in ${\sigma }_{pooled}$, rather than being associated with (a lack of) effect variation in mean differences.

If both ${CV}_{{\sigma }_{pooled}}$ and ${CV}_{MD}$ are nonzero, relative heterogeneity ${CV}_{d}$ is additionally affected by any non-zero covariation between ${\sigma }_{pooled}$ and$MD$. The scenario in Fig. 6b, for example, implies that larger unstandardized effects are associated with smaller${\sigma }_{pooled}$, resulting in a negative correlation ${r}_{MD,{\sigma }_{pooled}}$ and an increase in${CV}_{d}$, as Eq. (1) demonstrates. Whenever ${CV}_{{\sigma }_{pooled}}$ and ${CV}_{MD}$ are nonzero, a negative (or zero) correlation ${r}_{MD,{\sigma }_{pooled}}$ implies relative heterogeneity to be larger in$d$, while a positive correlation can also imply larger relative heterogeneity in$MD$. The correlation could help us to identify meta-analytic data sets with systematic differences in the distributions of the two effect sizes. Even in a scenario where the amount of heterogeneity is similar for ${CV}_{MD}$ and${CV}_{d}$, ${CV}_{{\sigma }_{pooled}}$ could be nonzero but masked by a positive correlation${r}_{MD,{\sigma }_{pooled}}$. Therefore, moderator analyses could still be affected by differences between the distributions of $MD$ and$d$.

There are, of course, other and more common relative heterogeneity measures, like ${I}^{2}$ and ${H}^{2}$ (e.g., Higgins & Thompson, 2002). But both depend on an estimation of heterogeneity and sampling variability, and would require us to make additional assumptions about the latter. Still, they provide important information on the general signal-to-noise ratio in the data, the amount of variability that is attributed to systematic differences between studies rather than to sampling error. If, for example, the respective ${I}^{2}$ is close to zero (or ${H}^{2}$ close to 1), we should refrain from generalizing estimates of the heterogeneity, even when $\tau$ or $CV$ are large. In the following section, we explore heterogeneity in multi-lab data using the umbrella, the correlation ${r}_{MD,{\sigma }_{pooled}}$, and all three of these relative heterogeneity measures.

### Heterogeneity in multi-lab data

Here, we explore the relationship between the restrictions of rating scales and heterogeneity estimates for $MD$ and $d$ of the same data set. We selected four data sets of non-null effects from Many Labs 1 (ML1; Klein et al., 2014) and Many Labs 2 (ML2; Klein et al., 2018), two of the largest multi-lab replication efforts within psychology. Our data selection (more information in the following section) is not representative and serves illustrative purposes, rather than a basis for generalization. We only considered multi-lab projects that ran direct replications, implying that expected heterogeneity estimates from their data are rather at the lower end of what we could observe for each effect. Additionally, manipulating the treatment strength across replications, for example, would (in most cases) imply additional effect heterogeneity. The restrictions of the umbrella could have more severe implications in conceptual replications than in the direct replications we present.

#### Data

We sourced our data from a collection of data sets from such multi-lab replications that we created as a research group, the DRIPHT Repository (https://osf.io/g2fzq/). The file with all data sets is available under https://osf.io/6pw3s/. We considered only direct replication multi-labs (like multi-lab registered replication reports and Many Labs projects) to make sure that the included data sets had the same design and similar sample sizes. Further, we selected only studies that used a single rating scale to measure the dependent variable and for which the standardized meta-analytic effect was at least small, $d>0.2$ according to convention (Cohen, 1992). Through these criteria, we aimed to identify data sets that could be affected by the suspected deviations. This left us with eight data sets which we initially plotted within their respective umbrella and from which we selected one, our first data set, that seemed unaffected by the restrictions and three for which the group results produced a pattern that could be affected by the restrictions. We selected four multi-lab data sets from two Many Labs projects: Data Sets 1 and 3 are replications of Lorge and Curtiss (1936) and of Oppenheimer et al. (2009) from ML1, the latter of which investigates the sunk cost effect, which originated in early decision research (e.g., Arkes & Blumer, 1985; Knox & Inkster, 1968; Thaler, 1985; Tversky & Kahneman, 1974). Data from both projects is openly available through the Open Science Framework (OSF). Data Sets 2 and 4 are replications of Hsee (1998) and of Knobe (2003) from ML2. Participants in Data Set 1 received a quote, which, depending on the experimental condition, was attributed to either a liked or a disliked individual. They were then asked to rate their agreement with the quote. Participants in Data Set 2 received a vignette that asked them to imagine receiving a goodbye gift from a friend. In one condition, that gift was a relatively expensive scarf within the presented price range of scarves, while in the other condition, it was a coat that was slightly more expensive than the scarf but very cheap compared to other coats. Subsequently, they rated the generosity of the gift. Participants in Data Set 3 received a vignette in which they were asked to imagine having tickets for a football game on a day that happened to be freezing cold. Depending on the experimental condition, they were asked to imagine either not having paid or having paid for that ticket and were subsequently asked to indicate their likelihood of attending the game. Participants in Data Set 4 received a vignette in which the vice president of a company accepted the side effects of a policy they would implement. Depending on the experimental condition, the side effects were either helpful or harmful to the environment. Subsequently, they rated the perceived intentionality of these side effects.

#### Analyses

We ran all our analyses in R (R Core Team, 2021). In a first step, we created an umbrella plot with group means and sample standard deviations (with $n$ in the denominator, not $n-1$) for each data set and inspected the respective pattern. Then we calculated the Pearson correlation coefficient and Spearman’s rank correlation coefficient, ${r}_{Pearson}$ and ${r}_{Spearman}$, between the $MD$ and ${s}_{pooled}$ using the *confintr* package (Mayer, 2023). We calculated the Spearman correlation as a non-parametric measure of the association, in case the relationship is nonlinear. These correlations are only proxies for ${r}_{MD,{s}_{pooled}}$ from Eq. (1), because they are affected by sampling error. Still, the larger the absolute values of ${r}_{Pearson}$ and ${r}_{Spearman}$, and the smaller their confidence intervals, the more likely the association is to affect effect heterogeneity in $d$. Finally, we ran meta-analyses of $MD$ and $d$ using the *metafor* package (Viechtbauer, 2010) with the residual maximum likelihood (REML) estimator (for a comparison of estimators, see Hönekopp & Linden, 2022) and report three relative heterogeneity measures: $CV$, ${H}^{2}$, and ${I}^{2}$. Borenstein et al. (2010) describe ${I}^{2}$ as the proportion of absolute effect heterogeneity to the total dispersion in the observed outcomes, a signal-to-noise ratio, which results in values from $0\%$ to $100\%$. ${H}^{2}$, on the other hand, is the ratio of the total dispersion to the amount of sampling variability. Thus, homogeneity results in ${H}^{2}=1$, with larger values as heterogeneity increases. A heterogeneity measure that does not depend on the sampling variability is the previously introduced coefficient of variation, $CV=\frac{\tau }{\mu }$, which standardizes absolute heterogeneity $\uptau$ on the mean of the distribution $\mu$. ${CV}_{d}=\frac{1}{3}$, for example, means that the standard deviation ${\uptau }_{d}$ fits three times between the observed meta-analytic mean and a null effect—almost all true effects from such a distribution would have the same sign. Additionally, we report the meta-analytic mean, tau, and the *p* values of the test for residual heterogeneity, $QEp$. We interpreted the *p* values for conventional significance thresholds $\alpha \le .05$ and $\alpha \le .01$ to check whether this classification is affected by the choice of effect size in our examples. Code and data to reproduce all our analyses are available through the associated OSF repository (https://osf.io/vc8u6/).

#### Umbrella plots

**Fig. 7:**
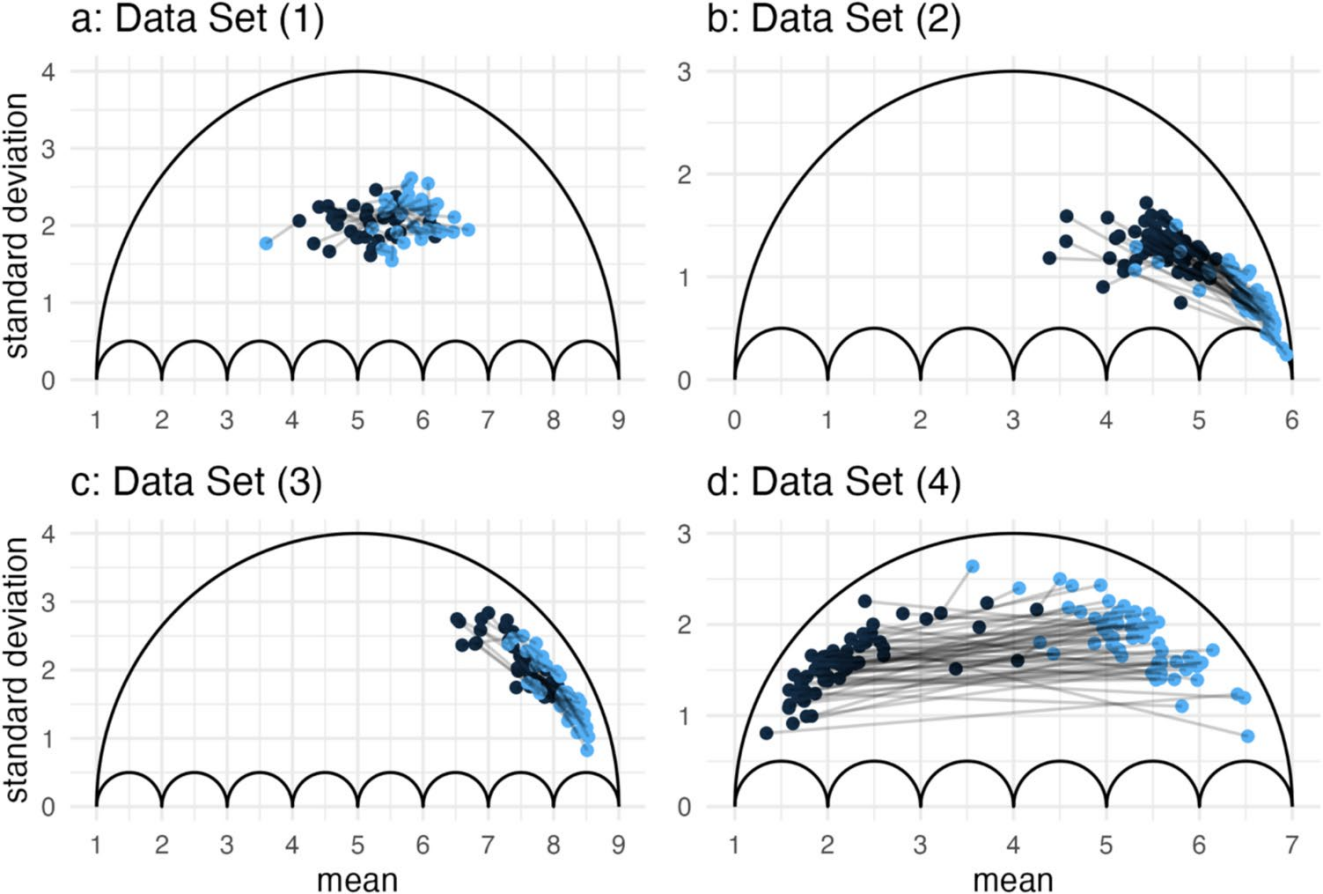
Plots depicting the outline of the umbrella for a scale from $0$ to $6$. We additionally present group means and standard deviations for Data Sets 1–4 within each umbrella. Plots **a** and **c** report nine-point scales, and plots **b** and **d** seven-point scales. The folder *Shiny_Umbrellas_Data* in the OSF repository of this article (https://osf.io/vc8u6/) contains the four aggregated data sets as csv files, which can be uploaded to the *Shiny Umbrellas* application to recreate these plots: https://www.apps.meta-rep.lmu.de/shiny_umbrellas/

Figure 7 depicts umbrella plots for multi-lab Data Sets 1–4, showing the outcomes of the control and treatment groups (black and blue points) per replication. The group results in Fig. 7a are scattered around the center of the umbrella; it seems unlikely that the scale induced differences between unstandardized and standardized effects. The results depicted in the remaining umbrellas (Fig. 7b–d), on the other hand, are scattered much closer to the respective outline. While the control group results (black) in Fig. 7b were quite far from the outline, the treatment group means approached the scale’s limit, and the standard deviations approached their minimum. There seem to be strong ceiling effects in some of the treatment groups, where most participants chose the highest integer on the scale. Here, it seems more likely that we would find differences in meta-analytic heterogeneity for $MD$ and $d$. Figure 7c produced a pattern consistent with our argument from Fig. 6b: The proximity of treatment and control group results to the upper outline was quite consistent across means. The treatment seemed to induce differences between experimental conditions, but responses within each group remained consistently polarized. Nonetheless, the standard deviations decreased toward the end of the scale, calling into question whether they can still be interpreted without considering their mean-specific restrictions (e.g., Taylor et al., 2023). If the changes in standard deviations do not represent meaningful changes to the variability, standardized mean differences could also misrepresent the treatment effects. Finally, the group means in Fig. 7d scattered across most of the range of the scale and toward the upper limit of possible variability. Some of the effects were very large, with both groups seemingly affected by the restriction to variability at the scale’s limits. Around its center, we found effects that were quite small, some with rather large standard deviations.

In this data set, increasing mean differences are associated with decreasing standard deviations, implying heterogeneity in $MD$—and even more in $d$.

#### Correlations

Table 2 presents the results for correlations ${r}_{Pearson}$ and ${r}_{Spearman}$. The correlations for Data Sets 1 and 2 were close to zero and their confidence intervals overlapped the null. We would expect this result for Data Set 1, from inspecting Fig. 7a, but for Data Set 2 it is more surprising. The curious dispersion within the umbrella in Fig. 7b does not seem to affect the correlation. Despite the scatter within the umbrella, the variation in either ${s}_{pooled}$ or $MD$ could be too low for a correlation between the two, or there is in fact no covariation. If the meta-analysis of $MD$ reports homogeneous effects, all heterogeneity in $d$ would be induced by that of the standard deviation, while very similar levels of heterogeneity in unstandardized and standardized effects would imply that the standard deviations do not contribute to heterogeneity. The correlations of Data Set 3 had confidence intervals that only slightly overlapped the null, indicating a somewhat stronger relationship between ${s}_{pooled}$ and $MD$ that could affect effect heterogeneity. Because the correlations are positive, relative heterogeneity may be smaller in $d$ than in $MD$. The confidence intervals for Data Set 4 were the narrowest of the four, and the correlation was very large and negative. In line with our expectations from Fig. 7d, the correlation suggests a considerable inflation of relative heterogeneity in $d$.

**Table 2:** Correlations and confidence intervals

Data Set	${r}_{Pearson}$	$LL$	$UL$	${r}_{Spearman}$	$LL$	$UL$
1	0.12	− 0.21	0.43	0.13	− 0.23	0.45
2	− 0.09	− 0.34	0.17	0.01	− 0.26	0.27
3	0.27	− 0.07	0.55	0.30	0.00	0.56
4	− 0.78	− 0.86	− 0.66	− 0.82	− 0.90	− 0.70

#### Effect heterogeneity

Here, we report the meta-analytic results for $MD$ and $d$ (Table 3) and relate them back to our expectations from the umbrella plots (Fig. 7) and the correlations (Table 2). Generally, there was considerable effect heterogeneity across data sets and effect sizes; the *p* value of the test for heterogeneity was smaller than any conventional alpha, except for Data Set 3. For Data Sets 1 and 2, the differences in relative heterogeneity were small across measures and consistent within each data set: relative heterogeneity in $MD$ was larger in Data Set 1 and smaller in Data Set 2. These differences are in line with the expectations from the signs of ${r}_{Pearson}$ and ${r}_{Spearman}$ in Table 2. Still, the differences between relative heterogeneity of $MD$ and $d$ are practically negligible. In both data sets, heterogeneity in $d$ is overwhelmingly induced by heterogeneity in $MD$ rather than by that in ${s}_{pooled}$. The positive correlations for Data Set 3 prompted us to assume relative heterogeneity to be smaller in $d$. But this data set appears to be an example where ${r}_{Pearson}$ and ${r}_{Spearman}$ do not represent ${r}_{MD,{s}_{pooled}}$ well, because Table 3 reports almost no heterogeneity in $MD$. If the unstandardized effects do not vary (after removing sampling error), they cannot covary with ${\sigma }_{pooled}$ either. Thus, heterogeneity in $d$ is induced by that of ${s}_{pooled}$, as demonstrated in Eq. (2). While this could potentially lead to misinterpretations in a moderator analysis, the issue may not be practically relevant for this data set. Relative heterogeneity measures ${H}^{2}$ and ${I}^{2}$ imply that most of the variation is attributed to sampling error, and the test for heterogeneity would not be significant at any conventional alpha. The results for Data Set 4, on the other hand, are fully in line with our expectations derived from the respective umbrella and correlations. Relative heterogeneity ${CV}_{d}$ was about $1.5$ times greater than ${CV}_{MD}$, as was also the case for ${H}^{2}$. Both ${H}^{2}$ and ${I}^{2}$ indicate that most of the variation was attributed to effect heterogeneity. Irrespective of choosing $MD$ or $d$ for the meta-analysis of this data set, we would conclude that there is considerable effect heterogeneity. However, an evaluation of the amount of heterogeneity, as well as moderator analyses, is much more likely to be affected by the choice of effect size.

**Table 3:** Meta-analytic results and relative heterogeneity measures

Data Set	ES	$\mu$	$\uptau$	$CV$	${H}^{2}$	${I}^{2}$	$QEp$	*df*
(1)	MD	0.651	0.365	0.560	2.241	55.4	.000	35
(1)	d	0.309	0.164	0.530	2.085	52.0	.001	35
(2)	MD	0.903	0.222	0.246	2.367	57.7	.000	58
(2)	d	0.830	0.226	0.272	2.484	59.7	.000	58
(3)	MD	0.583	0.046	0.079	1.022	2.2	.306	35
(3)	d	0.293	0.050	0.170	1.101	9.2	.422	35
(4)	MD	3.184	0.854	0.268	9.251	89.2	.000	60
(4)	d	1.897	0.783	0.412	14.560	93.1	.000	60

## General discussion

Means and standard deviations based on rating scale data are systematically restricted, as umbrella plots demonstrate (e.g., Heathers et al., 2018; Taylor et al., 2023). Our formalization based on the Bernoulli distribution gives us the lowest and highest possible sample standard deviations (or variances) for any particular mean on a given rating scale—an outline of the umbrella. We used that formalization to illustrate how the relative position of experimental group means within a scale relates to statistical power of Welch’s *t*-test. Further, we provided arguments and examples of response patterns that can lead to deviations in meta-analytic heterogeneity of unstandardized and standardized mean differences. The following discussion elaborates on implications of the observed restrictions for power analyses and heterogeneity analyses. We comment on the role of study design in facilitating meta-analytic deviations between unstandardized and standardized effects, briefly discuss alternative models to analyze rating scale data, and point out additional use cases of our umbrella formalization.

### Power analysis

Usually, we would interpret means as measures of central tendency and standard deviations as measures of dispersion, but in rating scale data, these are not independent of each other. On the contrary, the restrictions to means and standard deviations rather facilitate specific patterns of heteroscedasticity. Therefore, we recommend applying methods to rating scale data that do not rely on an assumption of homogeneity of variance, such as Welch’s *t*-test, to appropriately control type 1 error rates (see for example Delacre et al., 2017). Still, even though Welch’s *t*-test is designed to deal with heteroscedasticity, its application to rating scale data still requires careful consideration of the interpretability of its power. We demonstrated that statistical power is related to the position of group means within the umbrella. The same unstandardized effect has a smaller associated range of standardized effects and power at either limit of the scale than at its center.

Despite the interpretational challenges, we can also use the restrictions of the scale to our benefit: If researchers can point to the smallest effect of interest in its original units, the umbrella formalization helps to identify the largest possible standard deviation for that effect. The combination yields the smallest standardized effect of interest that allows for a maximally conservative estimate of the required sample size while also saving resources by excluding unrealistically large standard deviations. As we demonstrated in Fig. 5a, any effect $MD>1$ cannot be associated with $d<\frac{1}{3}$ on any seven-point scale, for example.

Further, when we aim to replicate a study, we may want to create an umbrella plot with group means and standard deviations of the original to check if they are close to the outline of the umbrella. That information can help us to evaluate how likely we may be to observe similar patterns in the data and to calibrate a priori power analyses. The R code for the umbrella is coded as a function in an individual script provided on GitHub, and we, of course, invite the reader to source, use, and adapt that function (https://github.com/JensFuenderich/Umbrella_RatingScales).

### Heterogeneity analysis

We demonstrated that heterogeneity in $d$ can be inflated or deflated, in comparison to that of $MD$. Specific designs can make either direction more likely. Designs like that of Knobe (2003), Data Set 4, treat both groups to maximize the effect rather than having a control and a treatment condition. Such designs inflate heterogeneity in $d$ by creating a negative correlation between $MD$ and ${s}_{pooled}$, the numerator and denominator of $d$. Working with rating scales requires us to be aware of these potential differences between unstandardized and standardized effects. We encourage researchers to specify which effect size relates more closely to their verbal hypothesis—$MD$ or $d$. This becomes crucial when we aim to identify moderators and are faced with a situation where the distributions of $MD$ and $d$ differ. If we aim to explain heterogeneity in $d$, for example, we may not be fully able to do so if it was inflated by the scale.

Patterns like those in Fig. 7b–d, Data Sets 2–4, also pose the question to what degree these results are related to the choice of scale. How would they change if we shortened or lengthened the scale? How would changes to the rating scale alter the measurement of the latent construct? We urge researchers to cautiously handle generalizations of effect heterogeneity when the results are as closely tied to the restrictions of a specific scale (length) as they are in Fig. 7b–d. This note of caution extends to the context of meta-scientific aggregations, where $d$ is often used to compare meta-analytic results across various designs and effects. The articles accompanying the Many Labs projects, for example, present kernel density estimates using standardized effect sizes for all replicated designs. Making such comparisons across designs and scales may produce misleading interpretations of the heterogeneity when some of the heterogeneity estimates and underlying effect sizes are closely related to the respective scale.

### Study design and effect heterogeneity

The way that means and standard deviations of experimental groups scatter within the umbrella is influenced by the size of the induced effects. While a control condition would generally remain unaffected by any treatment, positive effects push the treatment group mean towards the right corner of the umbrella. However, the replications of Knobe (2003) are vignette studies, in which the manipulation in one group is a description of harmful, and that of the other a description of helpful side effects. Contrary to our expectation of a typical experimental design, we do not find a neutral control condition. Instead, there are two treatment conditions that aim to push participants’ responses on the dependent variable in opposite directions, as depicted in Fig. 7d. The fact that they scatter close to the upper edge of the umbrella indicates strong polarization within the samples and groups. Since both experimental conditions are affected by a treatment and produce this polarization, both groups’ standard deviations are increasingly restricted for larger effects. A large negative correlation between $MD$ and ${s}_{pooled}$ (see Table 2) could be a more common characteristic of this type of design and increase the likelihood of deviations between the heterogeneity of unstandardized and standardized effects. Further, any moderator may affect both experimental conditions of such a design, either in the same or in opposite directions. In conclusion, adequate explanations of effect heterogeneity need to consider decisions around measurement and study design just as much as the theory and previously overlooked moderators.

### Alternative approaches

The tools and comparisons that we used and introduced throughout this article do not require additional training or novel methodology to evaluate the relationship between a rating scale and a study’s results. Nonetheless, there may be statistical approaches that could improve the way we handle rating scale data. The limitations of the umbrella reduce the chances that an assumption of variance homogeneity (between experimental conditions and between replications) is sensible. We can use location-scale models to aggregate data and to define predictors of variance heterogeneity (McNeish, 2021; Rodriguez et al., 2023; Viechtbauer & López-López, 2022). Alternatively, we could apply ordinal models for calculating effect sizes and appropriate standard errors for rating scales. Frank & Heene (2024) meta-analyze effect estimates from ordinal models (Bürkner & Vuorre, 2019) and compare the resulting effect heterogeneity to estimates from linear models. Their preprint contains Data Sets 1–4 from our analyses, allowing a comparison to our heterogeneity estimates. The ordinal effect estimates of Data Set 1 are about as heterogeneous as those of the linear model, with ${H}^{2}=1.99$ and $CV=0.51$. This is in line with the fact that the data points (the associated means and standard deviations) scatter around the center of the umbrella. The underlying distributions could be rather normal and therefore well represented by the linear model. For Data Sets 2 and 3, they are more heterogeneous in comparison to heterogeneity in $MD$ and $d$, with ${H}^{2}=1.39$ and $CV=0.33$ for Data Set 3, for example. In contrast, heterogeneity of the ordinal model for Data Set 4 is lower than that of $MD$ and $d$.

Arguments concerning the preference or superiority of linear versus ordinal models often revolve around the risk of linear analyses producing misleading results. Liddell & Kruschke (2018) illustrate this point with a metaphor: conducting a linear analysis on ordinal data and hoping the analysis results will not be affected is like driving drunk and hoping the car will not crash. In the context of this metaphor, the umbrella can be viewed as a (rough) sobriety test to detect patterns that are more likely to produce misleading results when analyzed with a linear model. Ordinal models and other non-parametric procedures will usually be more appropriate for analyzing data from a typical rating scale. Still, for the time being, meta-analyses of standardized effect sizes like $d$ are common within the literature, requiring us to be aware of their potential pitfalls. Again, we do not advocate for a specific model (for literature that does, see for example Bürkner & Vuorre, 2019; Liddell & Kruschke, 2018; Liu & Agresti, 2005). The goal of this article is to equip researchers with the knowledge and tools to understand the restrictions within which they operate when they apply the presented parametric analyses to rating scale data.

### Utilizing the umbrella

Finally, we want to point out potential use cases for our formalization of the umbrella outline, a few of which we have implemented in a Shiny application. One way to utilize the formalization is for developing alternative measures of agreement between ratings. The previously mentioned report by Taylor et al. (2023) contains observations of data from norming studies that are affected by the dependency between means and standard deviations (they reproduce patterns like the one in Fig. 7d, though in a non-experimental setting). This is an issue to their approach, because they interpret standard deviations as a measure of agreement between responses on the rating scale. These comparisons are typically made across different items with different means, which obscures their interpretability, as pointed out by Taylor et al. (2023). The umbrella outline could help create other measures of agreement or polarity: the relative position of the observed standard deviation (on the *y*-axis) between the minimum and maximum standard deviation attainable at that mean (on the *x*-axis). This would allow for a more consistent comparison of the agreement or polarity across samples with different means.

The other use cases we want to highlight are more closely related to the presented analyses, and available through *Shiny Umbrellas*, a Shiny app that is hosted openly available on servers of the LMU Munich: https://www.apps.meta-rep.lmu.de/shiny_umbrellas/. The first tab of the app, *Discrete Umbrella*, provides a (for computational reasons) very limited interface to simulate illustrations like that of Fig. 1 or the original from Heathers et al. (2018), which used a five-point scale and a sample of 10 participants. The second tab in *Shiny Umbrellas*, *Error Checking*, provides a computationally quick but, especially in smaller samples, less precise method than SPRITE (Heathers et al., 2018) for checking the congruence between information on a scale and the according means and standard deviations. The third tab uses the formalization to identify the largest standard deviation that a mean difference can be associated with on a given scale. That standard deviation can be used to identify nominal (!) a priori power of a test for a respective sample size, as the *Power* tab in *Shiny Umbrellas* does for Welch’s *t*-test. The implementation assumes equal sample sizes per experimental condition. The fourth tab, *Meta-Analysis*, allows users to investigate a meta-analytic data set of means and standard deviations by presenting it in the umbrella (see Fig. 7), and reports relative heterogeneity measures for unstandardized and standardized mean differences. The respective meta-analyses are implemented using the *metafor* package (Viechtbauer, 2010).

### Revisiting the rating scale and the generalizability of the umbrella approach

We introduced our formalization of the umbrella and its applications in the context of rating scales, which we defined as having an upper and a lower bound, consisting only of integers, and being applied in samples of finite size. This definition is not unique to rating scales. For example, if the dependent variable is the number of correct responses in a set of 10 questions, this is essentially equivalent to an 11-point rating scale ranging from 0 to 10. The restrictions and the relationship implied for means and standard deviations are the same as for the rating scale, making the umbrella and its formalization applicable to such scales as well. Any scale that shares these properties will necessarily fit this framework. This includes some borderline cases, such as counting rare events, for which the applicability of the umbrella depends on knowing the maximum count to expect. Additionally, the constraints on the variability—standard deviations or variances—described by the umbrella also hold for multi-item scales. This is the case, for example, when the dependent variable is defined as the mean of 10 five-point rating scales. Furthermore, our definition of a rating scale is by no means universal. Visual analogue scales, for example, are typically used as rating scales and, in line with our definition, have both a lower and an upper bound. Nonetheless, we could implement such a scale to be effectively continuous between these bounds. In terms of our formalization, this would imply that the upper limit of standard deviations remains identifiable, but the lower limit would not, as the scale is not limited to a finite number of discrete values.

## Limitations

We want to point out three limitations to the generalizability of our observations. The first is our choice of data sets: They are not randomly sampled, not representative of the psychological literature, and do not warrant any generalization. These four were merely interesting examples out of the eight that we identified as suitable for our analyses. The second is our focus on designs that use only a single rating scale for the dependent variable. When a dependent variable is calculated from multiple (rating scale) items, the potential consequences become more complex. But we assume even aggregated measures could produce similarly obscured heterogeneity estimates: If multiple items represent a single construct, results on these items are highly correlated. If participants in an experimental condition tend towards the scale limit in one item, other items should exhibit a similar trend. The third is our focus on replications that use the same scale. When the scale varies across studies, or within an aggregated measure, the issue becomes increasingly complex. It could still be informative to create groups of items or studies that share the same scale and to plot them within their respective umbrella. If the number of studies in a meta-analytic collection of conceptual replications is large enough, it may be informative to investigate the subgroups defined by the scale lengths and their respective heterogeneity.

## Conclusion

Means and standard deviations are commonly interpreted as independent measures of location and dispersion. There are, however, measures that increase the likelihood of a dependency between location and dispersion. We presented the case of single-item rating scale responses as an example that is prominently used in psychology and behavioral research. One solution is, of course, to move towards methods and models that are better at handling these restrictions (some of which we mentioned in our discussion). Sticking to more conventional models, as much of the literature still does, has often overlooked consequences, like the implication that an unstandardized effect $MD=1$ is associated with varying statistical power as a function of its position within the scale. Or that these restrictions can induce differences between meta-analytic results for unstandardized and standardized effects if the group means scatter across the measure.

A great deal of effort has been invested to evaluate if factors like questionable research practices (Anderson & Liu, 2023), samples and settings (Klein et al., 2018), or omitted moderators (Krefeld-Schwalb et al., 2024), but also the time of semester (Ebersole et al., 2016), pre-data-collection peer review (Ebersole et al., 2020), or original author involvement (Klein et al., 2022) affect replicability and effect heterogeneity. We would like to see more of that effort going into critical examinations of the interaction between common measures and methods, and how overlooked implications of such interactions contribute to the lack of cumulativeness in psychological research. The single-item rating scale is probably one of the more self-evident and extreme measures, but many of the standard assumptions may also be violated in other measurements like percentages, count data, or visual analogue scales. And there are, of course, other factors that affect our measures of location and dispersion. Standard deviations are also affected by range restrictions (Dahlke & Wiernik, 2020), measurement error variance (Wiernik & Dahlke, 2020), and differential responses (Kim & Seltzer, 2011). Taking our measures and their restrictions seriously can illuminate how our data interact with our statistical tools, how to exploit the limits of these measures (e.g., to adapt a priori power analyses or detect reporting errors), and even explain (some of the) replication failures and effect heterogeneity. We hope that our work is a contribution to move towards these goals.

## Data Availability

We did not create any new data, but used publicly available data from Many Labs 1 (https://osf.io/wx7ck/) and Many Labs 2 (https://osf.io/8cd4r/), which we sourced from a repository created by our project group (https://osf.io/g2fzq/).

## Code availability

The OSF repository for this article contains all data, graphics, and tables, and it directs users to the code, which is hosted on GitHub: https://osf.io/vc8u6/.

## Declarations

### Prior versions

https://osf.io/preprints/osf/7au4q_v1

https://osf.io/preprints/osf/7au4q_v2

https://osf.io/preprints/osf/7au4q_v3

https://osf.io/preprints/osf/7au4q_v4

https://osf.io/preprints/osf/7au4q_v5
